# Alcoholic Nostrum Knocked out [?]

**Published:** 1905-10

**Authors:** 


### Alcoholic Nostrum Knocked Out [?].

The United States Commissioner of Internal Revenue has issued the following order to all collectors:

"Among the various alcoholic compounds now on the market, advertised and sold as medicine under the name of whisky, bitters, tonics, cordials, etc., there are some that are composed chiefly of distilled spirits or mixtures thereof, without the addition of drugs or medicinal ingredients in sufficient quantities to change materially the character of the alcoholic liquor.

"The fact that these compounds during the existence of the statute imposing tax on proprietary medicines were without the necessity of investigation into their medicinal character by the terms of the law made subject to that tax, because they were held out to the public as medicines, does not afford grounds for relieving the manufacturers from special tax as rectifiers and liquor dealers, or dealers therein from special tax as liquor dealers, under the provisions of Section 3244, Revised Statutes, and amendments.

"It is held that the statute requires the exaction of this special tax from the manufacturer of every compound composed of distilled spirits, even though drugs are declared to have been added thereto, when their presence is not discoverable by chemical analysis, or it is found that the quantity of drugs in the preparation is so small as to have no appreciable effect on the alcoholic liquor of which the compound is mainly or largely composed.

"The same ruling applies to every alcoholic compound labeled as a remedy for diseases and containing, in addition to distilled spirits, only substances or ingredients which, however large their quantity, are not of a character to impart any medicinal quality to the compound; but where substances undoubtedly medicinal in their character are combined with whisky or other alcoholic liquor, and are used in sufficient quantities to give a medicinal quality to the liquor other than that which it may inherently possess, such compound is, of course, not to be included in this ruling.

"The question in such case, arising under the terms of this circular, will be determined by this office, not merely upon examination of the formula submitted by the manufacturer of the compound, but upon result of the analysis made in the chemical laboratory here of samples obtained in the open market and sent in by the local internal revenue officers and agents.

"The ruling of these compounds in the fourth paragraph of circular No. 608 (Treasury Decisions, 1901, Vol. 4, p. 210), 'that if they are composed of spirits in combination with drugs, herbs, roots, etc., and are held out as remedies for diseases stated in labels on the bottles they are to be regarded as medicines until the facts are ascertained, as to the purpose they are usually sold or used, show them to be beverages, and until such facts are obtained druggists and merchants who sell these compounds in good faith as medicines only are not to be called on to pay special tax as liquor dealers on account of such sales,' is hereby revoked.

"But in order that no injustice may be done to these druggists and merchants who, without holding special tax stamps as liquor dealers, now have in stock these compounds for sale as medicines, this circular will not be put into effect until December 1, 1905.

"Collectors will, however, immediately upon receipt of this circular send out notice to all druggists and merchants dealing in proprietary medicines in their districts who do not hold the re-

IN ANY CONDITION

of impaired health characterized
by a tendency to general
exhaustion, the patient who takes

GRAY'S Glycerine TONIC Comp.

regularly will eat more,
sleep better, be less nervous,
have more strength, be less dragged out

THE PURDUE FREDERICK CO.,
298 Broadway, New York quisite special tax stamp as liquor dealers, that on and after December 1, 1905, they will be required to pay special tax for selling the alcoholic compounds coming within the ruling now promulgated, even when they sell them in good faith for medicinal use only, never selling them as beverages, nor selling them knowingly to those buying them for use as beverages."

